# Multivariate approach to quantitative analysis of *Aphis gossypii* Glover (Hemiptera: Aphididae) and their natural enemy populations at different cotton spacings

**DOI:** 10.1038/srep41740

**Published:** 2017-02-09

**Authors:** José B. Malaquias, Francisco S. Ramalho, Carlos T. dos S. Dias, Bruno P. Brugger, Aline Cristina S. Lira, Carlos F. Wilcken, Jéssica K. S. Pachú, José C. Zanuncio

**Affiliations:** 1 Universidade de São Paulo (USP), Escola Superior de Agricultura “Luiz de Queiroz”, ESALQ, Departamento de Entomologia, Piracicaba, São Paulo, Brazil; 2 Unidade de Controle Biológico, Embrapa Algodão, Av. Osvaldo Cruz, 1143, Campina Grande-PB, CEP 58428-095, Brazil; 3 Universidade de São Paulo (USP), Escola Superior de Agricultura “Luiz de Queiroz”, ESALQ, Departamento de Ciências Exatas, Piracicaba, São Paulo, Brazil; 4 Universidade Federal de Viçosa, Departamento de Entomologia, Viçosa, Minas Gerais, Brazil; 5 Faculdade de Ciências Agronômicas, Departamento de Proteção Vegetal, Botucatú, São Paulo, Brazil; 6 Universidade Federal de São Carlos, UFSCAR, Departamento de Agroecologia e Desenvolvimento Rural, Araras, São Paulo, Brazil

## Abstract

The relationship between pests and natural enemies using multivariate analysis on cotton in different spacing has not been documented yet. Using multivariate approaches is possible to optimize strategies to control *Aphis gossypii* at different crop spacings because the possibility of a better use of the aphid sampling strategies as well as the conservation and release of its natural enemies. The aims of the study were (*i*) to characterize the temporal abundance data of aphids and its natural enemies using principal components, (*ii*) to analyze the degree of correlation between the insects and between groups of variables (pests and natural enemies), (*iii*) to identify the main natural enemies responsible for regulating *A. gossypii* populations, and (*iv*) to investigate the similarities in arthropod occurrence patterns at different spacings of cotton crops over two seasons. High correlations in the occurrence of *Scymnus rubicundus* with aphids are shown through principal component analysis and through the important role the species plays in canonical correlation analysis. Clustering the presence of apterous aphids matches the pattern verified for *Chrysoperla externa* at the three different spacings between rows. Our results indicate that *S. rubicundus* is the main candidate to regulate the aphid populations in all spacings studied.

Changing the conventional cotton agroecosystem structure is a common practice in Brazilian crops, and a reduced spacing between rows is one of the most commonly implemented changes. The “ultra-narrow row cotton” (UNRC) technology, i.e., cotton grown in ultra-narrow rows, has the potential to optimize cotton yield and/or phenology, to reduce costs by increasing yield per cultivated area^1^. In Brazil, especially in the Middle West, narrow row spacing in cotton favors the possibility of growing two crops within a season and its use in crop rotation^2^. In another hands it is possible that dense planting of a plant species does not always increases yield because plant density also affects the interactions among plants, insect pests, plant pathogens, natural enemies, and volunteer plants. The relative importance of such interactions changes according to the farming system. So far, there is no information about the effect of UNRC technology on arthropod population ecology

The faunal sampling in agricultural systems allows for the efficiently classification of populations to support decision-making processes, such as spraying products and/or release of natural enemies. It is necessary to identify the population dynamics of the arthropods found in an agricultural system before a sampling plan is put into practice. The ecological interactions involving individuals of a particular species and their natural enemies in a specific habitat are highly relevant variables in determining the variation in population densities^3^ as well as to effectively elucidate the patterns of association among populations of different species^4^. Studies have been conducted in recent years to improve ecological pest management, and especially the management of *Aphis gossypii* Glover (Hemiptera: Aphididae) in cotton farming systems under different planting configurations^5^,^6^,^7^. *Aphis gossypii* is considered the main sucking pest in Brazilian cotton culture because it reduces crop yield about 37%^6^. It attacks plants and causes direct damage by sucking the plant phloem as well as indirect damage by transmitting viruses and the excessive excretion of carbohydrates derived from the phloem sap^8^. This excretion may promote the occurrence of fungi, which inhibit the photosynthetic activity of the plant, thus resulting in chlorosis and, consequently, in yield losses^8^.

Multivariate analyses are valuable tools when in studies regarding the interactions among many species of organisms from different trophic levels. They allow the exploration of structural links between a great number of variables and enable the assessment and prediction of effective biological control agents; they also provide a means for habitat classification and the identification of features of the crops that are associated with the establishment, efficiency and population or community structure of natural enemies^9^. They may also be used to select biocontrol agents that are able to colonize the crop of interest to the farmer^10^. Such approaches as such as principal components, discriminant analysis, multidimensional scaling and multivariate variance analysis have been used in studies about of the abundance of herbivores and the richness of natural enemies. They allow for the comparison of typical arthropod community levels among different agroecosystem management types^11^.

Multivariate analyses also reduce a large number of variables into a few dimensions with minimal information loss to enable the detection of key similarities, associations, and correlation patterns among variables^11^. Based on previous studies, it is possible to affirm that the effect of agroecosystem structure on population of multiple species^12^ and on tritrophic interactions^13^ may be assessed with use of the Multivariate Analysis of Variance. With principal component analysis it is possible to identify the dominance of pests and their natural enemies and the pattern of occurrence at different growth stages of plant species^14^. The correlations between the dominant insect pests and the dominant natural enemies may be evidenced by canonical correlation analyses^14^, because canonical correlation analysis studies the linear relation between two groups of standardized variables^15^, like one group build with pest and another group made by natural enemies, attempting to find the pair of linear combinations (canonical variables) of each group with the maximum linear correlation^16^,^17^. In addition stepwise regression may help in predicting which potential natural enemies may regulate the population levels of the pest and the interactions between the species^18^ because is interactively constructed by a sequence of regression models through the addition or removal of variables at each stage^19^. Analyzes of the connections between organisms with focus in community ecology has been considered clustering coefficient^20^, and based on presence of arthropods clustering techniques allow analyzing co-occurrence and visualize closer or more distant occurrences between the pests and their natural enemies to be compared^4^.

The use of multivariate statistics should be encouraged in ecological studies because ecological issues are often multivariate and involve a great number of interactions among response variables^21^. It is not surprising that the cultural modifications of the cotton cultivation system can alters the pattern of insects occurrence, consequently it is necessary make alterations on the samplings programs and on tactics of release and conservation of natural enemies in biological control programs. There has been no record of the seasonal population dynamics of cotton aphid and their natural enemy species among different plant spacing. Thus, the aims of the current study are: (*i*) to characterize the occurrence on the apterous and alate forms of *A. gossypii* and their natural enemies based on principal components, as well as (*ii*) to analyze the correlations among groups of aphids (apterous and alate) and natural enemies, (*iii*) to identify if the natural enemies responsible for regulating the *A. gossypii* population are different among the spacings studied, and (*iv*) to evaluate patterns of similarity in the occurrence of both aphids and their biocontrol agents at different spacings of the cotton crop in two different seasons (2013 and 2014).

## Results

The multivariate statistical tests Wilks’ Lambda, Pillai’s Trace and the Hotelling-Lawley Trace showed significant differences among spacings (df = 14; P > F < 0.05), assessment time (df = 14; P > F < 0.05) and year (df = 14; P > F < 0.05) (Table 1) in terms of the occurrence of aphids and their natural enemies in the studied agricultural systems. However, the interactions of spacing × assessment time x year (df = 14; P > F > 0.05) and assessment time x year were not significant (df = 14; P > F > 0.05) (Table 1). On the other hand, the effect of the assessment time factor on the occurrence of aphids and their natural enemies in the studied agricultural systems depends on the assessment year (df = 14; P > F < 0.05) (Table 1).

The analysis of the consequences of the spacing x year interaction was carried out using contrasts and by individually comparing each assessment of aphid occurrence. The natural enemies of aphids in crop systems showed no differences in the occurrence of multiple variables between the assessments performed at 42 days in comparison to those carried out at 84, 105 and 112 days and between the assessments performed at 105 days in comparison to those carried out at 112 and 119 days in both 2013 and 2014. On the other hand, the variables that occurred at 35 days differed from those that occurred at 49, 56, 63, 98 and 112 days in 2013; however, there were differences in the occurrence of such variables at 35 days in comparison to the others in 2014, except for those that occurred at 42 and 112 days. These results showed that the fluctuation in the aphid population and that of its natural enemies is a function of the assessment time and depends on the cotton cultivation year, according to the multivariate perspective. By comparing the multiple variables between spacings using all the multivariate statistical tests listed in Table 1, it is possible to identify differences between the 0.40 and 0.80 m (F = 2.76; P > F = 0.0075) spacings and between the 0.80 and 1.60 m (F = 2.72; P > F = 0.0082) spacings. There was no significant difference between the 0.40 and 1.60 m spacings (F = 1.17; P > F = 0.3160).

**Figure 1:**
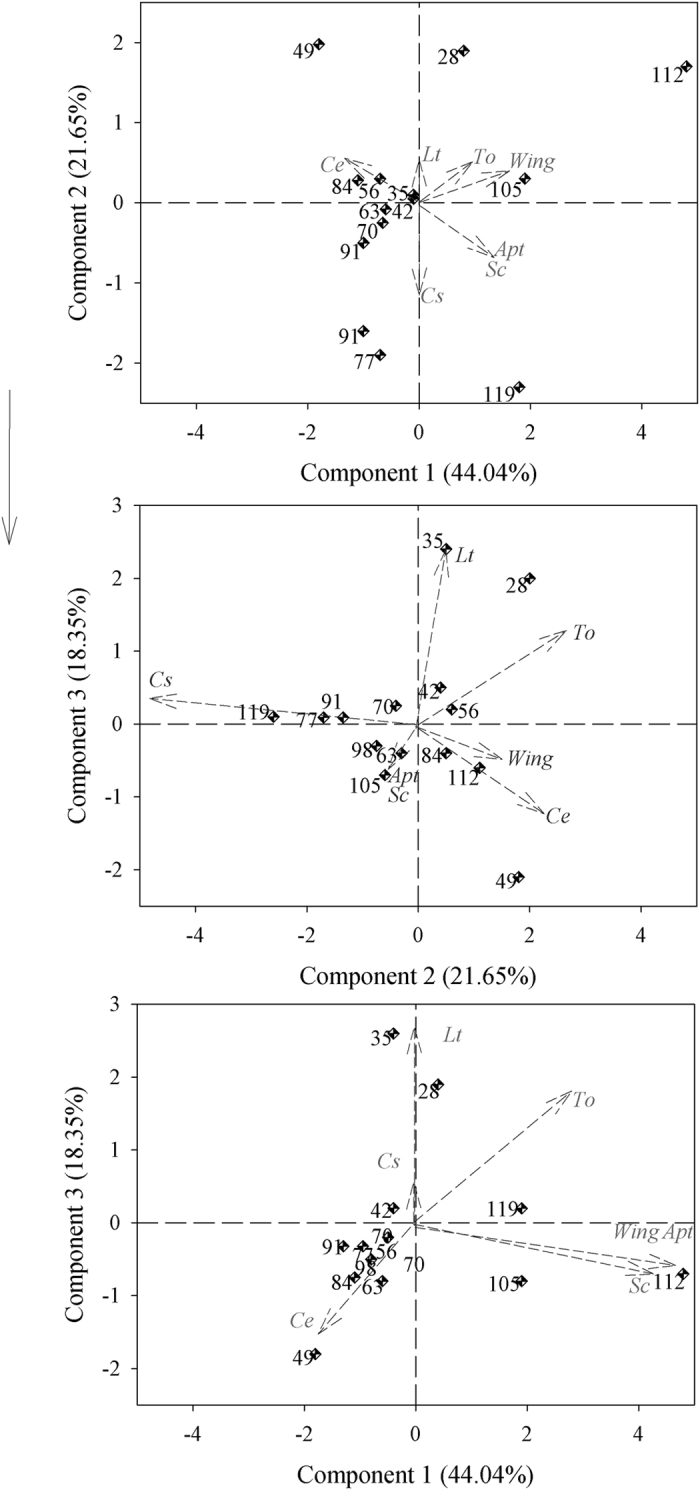
Biplots for the two aphid morphs: apterous (*Apt*) and alate (*Wing*), *Scymnus rubicundus (Sc*), *Toxomerus watsoni (To*), *Chrysoperla externa (Ce*), *Cycloneda sanguinea (Cs*) and *Lysiphlebus testaceipes (Lt*) on cotton grown at the 0.40 m row spacing. The dots represent the assessment time (days after plant germination), whereas the arrows represent the vector for each variable.

**Figure 2:**
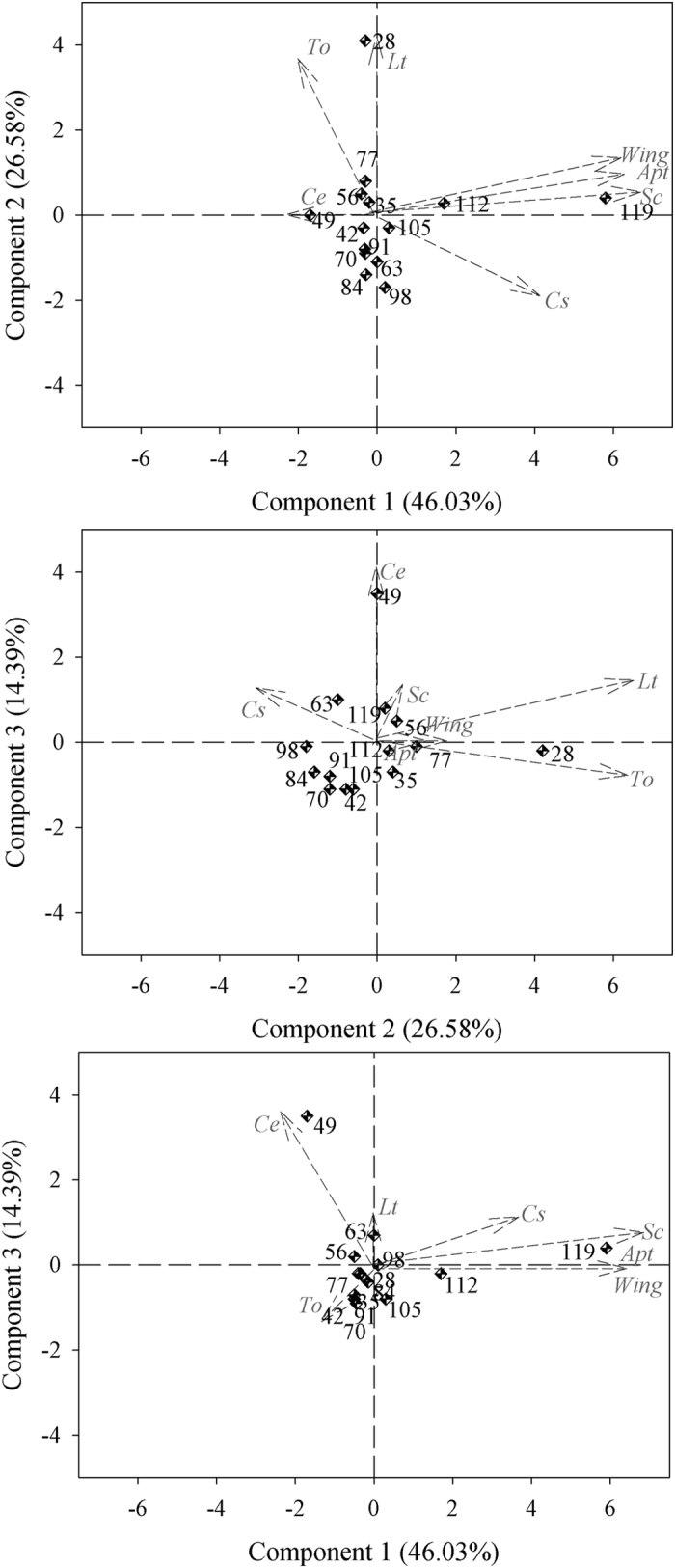
Biplots for the two aphid morphs: apterous (*Apt*) and alate (*Wing*), *Scymnus rubicundus (Sc*), *Toxomerus watsoni (To*), *Chrysoperla externa (Ce*), *Cycloneda sanguinea (Cs*) and *Lysiphlebus testaceipes (Lt*) on cotton grown at the 0.80 m row spacing. The dots represent the assessment time (days after plant germination), whereas the arrows represent the vector for each variable.

**Figure 3:**
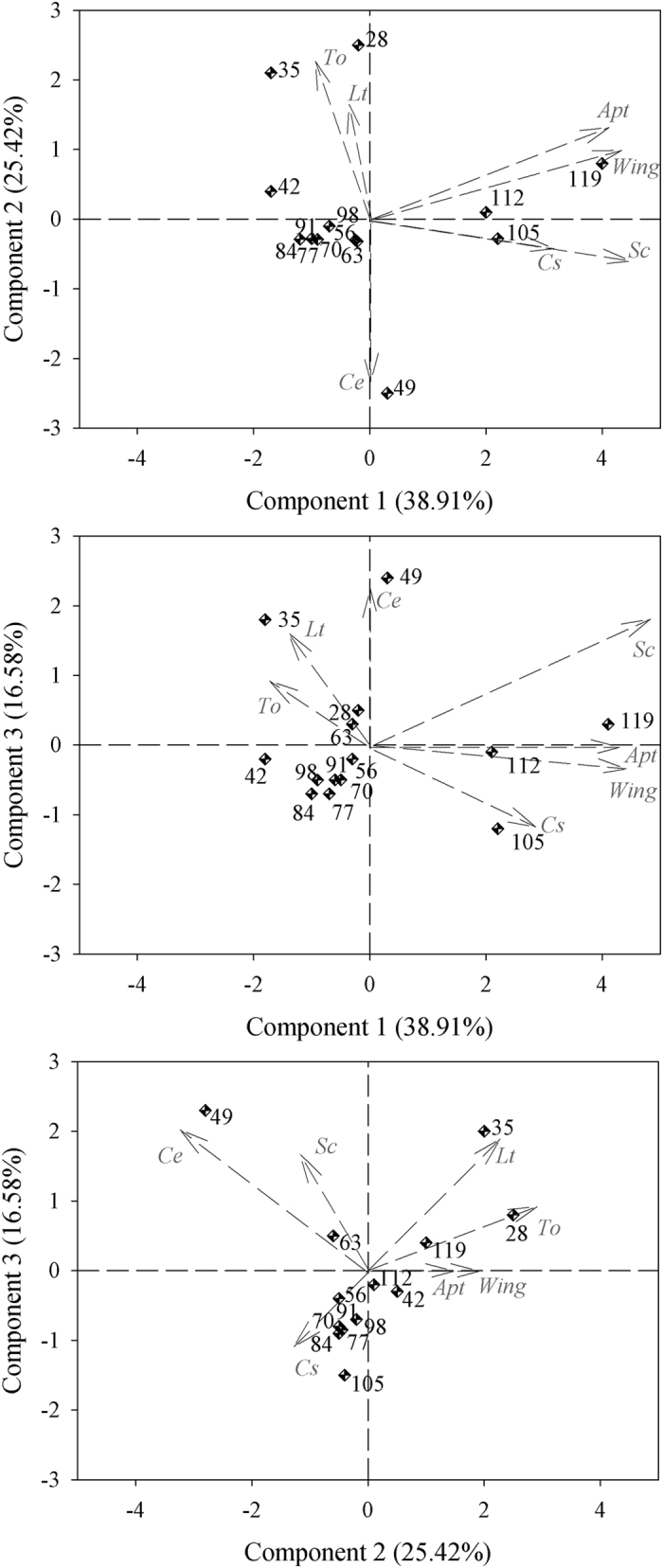
Biplot for the two aphid morphs: apterous (*Apt*) and alate (*Wing*), *Scymnus rubicundus (Sc*), *Toxomerus watsoni (To*), *Chrysoperla externa (Ce*), *Cycloneda sanguinea (Cs*) and *Lysiphlebus testaceipes (Lt*) on cotton grown at the 1.60 m row spacing. The dots represent the assessment time (days after plant germination), whereas the arrows represent the vector for each variable.

The results of the Pearson’s correlation analysis and the arrangement of the vectors in the biplots, especially those of the first components (Figs 1,2 and 3), show a high level of correlation between apterous and alate aphids at all spacings (Pearson’s r > 0.8000), whereas the most significant correlations with natural enemies occurred between the *Scymnus* (Pullus) *rubicundus* Erichson (Coleoptera: Coccinellidae) and both apterous and alate aphids; the correlations were higher than 0.6000 (Pearson’s r) for both types of aphids. The conventional spacing (0.80 m) showed correlations between *Toxomerus watsoni* (Curran) (Diptera: Syrphidae) and *Lysiphlebus testaceipes* (Cresson) (Hymenoptera: Braconidae) (Pearson’s r = 0.9127) and between *Cycloneda sanguinea* (Linnaeus) (Coleoptera: Coccinellidae) and *Scymnus (Pullus) rubicundus* (Fig. 3). Three components were selected for all spacing contingents on the eigenvalues of the correlation matrix according to the criterion by Kaiser^22^, which is based on the presence of components with eigenvalues greater than 1. The first principal component (PC1) is represented by the weighted average of the number of apterous (x1) and alate (x2) aphids and *S. rubicundus* (x5) beetles in the 0.40 m (0.5350 × 1 + 0.5339 × 2 + 0.5282 × 5), 0.80 m (0.5424 × 1 + 0.5181 × 2 + 0.5470 × 5) and in the 1.60 m spacings (0.5391 × 1 + 0.5472 × 2 + 0.4966 × 5), and explains 44.05, 46.03 and 38.91% of the total variation in the populations, respectively. The PC2 for narrow cotton (0.40) includes the contrast between the occurrence of *C. sanguinea* (x4) and the weighted averages of the occurrences of *T. watsoni* (x7) and *Chrysoperla externa* (Hagen) (Neuroptera: Chrysopidae) (−0.7482 × 6 + 0.4431 × 7 + 0.4348 × 4). The PC3 includes the contrast between the occurrence of *C. externa* and the weighted average of the occurrences of *L. testaceipes* and *T. watsoni* (−0.4855 × 4 + 0,7629 × 3 + 0.3526 × 7). These two components (PC2 and PC3) explain 21.64 and 18.35% of the total population variation, respectively.

Regarding the 0.80 m spacing, the PC2 includes the contrast between the occurrence of *C. sanguinea* and the weighted average of the occurrences of *L. testaceipes* (x3) and *T. watsoni* (X7) (−0.2962 × 6 + 0.6607 × 3 − 0.6544 × 7). *Lysiphlebus testaceipes* (x3) and *C. sanguinea* (x6) also have great participation in PC3 and the weighted average of the occurrence of *C. externa* (x4) (0.9560 × 4 + 0.1835 × 3 + 0.1739 × 6). For the 1.60 m spacing, the PC2 includes the contrast between the occurrence of *L. testaceipes* (x3) and the weighted average of the occurrences of *C. externa* (x6) and *S. rubicundus* (x5) (0.4734 × 3 − 0.5211 × 6 − 0.5643 × 5), whereas the PC3 includes the weighted average of the occurrences of the same natural enemies (0.6238 × 4 + 0.4944 × 3 + 0.4501 × 5). Components 2 and 3 (PC2 and PC3) explain 26.58 and 14.39% of the total population variation in the 0.80 m spacing, respectively, whereas these two components (PC2 and PC3) explain 25.42 and 16.58% of the total population variation in the 1.60 m spacing, respectively. Therefore, components 1, 2 and 3 (PC1, PC2 and PC3) explain 84.05, 87.01, and 80.91% of the variation in the 0.40, 0.80 and 1.60 m spacings between rows, respectively (Figs 2 and 3).

The biplot chart confirms the Pearson’s and canonical correlation analyses of all the studied spacing conditions because the occurrence of *S. rubicundus* was directly related to that of apterous and alate aphids. The highest occurrences of the apterous and alate forms of *A. gossypii* and of its predator *S. rubicundus* were concentrated in the last three assessments, i.e., 105, 112 and 119 days after the germination of cotton plants, with the only exception being 105 days after the germination of cotton plants in the 0.80 m spacing rows. An inverse pattern was found when comparing the distribution of the data on the occurrence of *T. watsoni* to that of the apterous and alate aphids in the 0.80 and 1.60 m spacings or to that of the *L. testaceipes* parasitoid in all spacings (Figs 1,2 and 3). The high correlations between *T. watsoni* and *L. testaceipes* and between *C. sanguinea* and *S. rubicundus* were also seen in the 0.80 m row spacing. In addition, these natural enemies were more abundant at the beginning of the development cycle of cotton plants (at 28 days) in the cases of *T. watsoni* and *L. testaceipes* and at the end of the crop cycle (at 112 and 119 days) in the cases of *C. sanguinea* and *S. rubicundus* (Figs 1,2 and 3).

There were canonical correlations between group V1 (apterous aphids + alate aphids) and group W1 (natural enemies) in all the studied spacings (Table 2). *Scymnus* (Pullus) *rubicundus* was the variable that most contributed to the formation of the canonical component 1. It was followed by *T. watsoni* and *C. sanguinea* in the 0.40 and 0.80 m spacings between rows, respectively, and by *L. testaceipes* and *C. externa* in the 1.60 m spacing between rows (Table 2).

In the stepwise multiple regression analysis (Table 3), the variables were selected using predictive models, which are able to estimate the occurrence of apterous and alate aphids. The mathematical models are −0.02 − 0.81x + 7.76y + 3.61z (alate, 1.60 m) (F_3,24_ = 37.41, P > F: 0.0001), 9.77 − 46.21x + 437.37y (apterous, 1.60 m) (F_2,26_ = 27.40, P > F: < 0.0001); 9.77 − 46.21x + 437.37y − 1.47z (apterous, 0.80 m) (F_3,24_ = 69.38, P > F: < 0.0001), 13.83 + 82.74x + 388.80y − 114.18z (alate, 0.80 m) (F_3,24_ = 113.80, P > F: < 0.0001); −0.19 − 1.84x + 9.47y + 11.97z (alate, 0.40 m) (F_3,24_ = 49.54, P > F: < 0.0001) and 3.70 + 413.08x + 93.71y (apterous, 0.40 m) (F_2,25_ = 127.90, P > F: < 0.0001). Based on the partial R^2^ values, it was observed that *S. rubicundus* (x1) (R^2^ > 0.4600) was the only independent variable found in all models (spacing/apterous/alate). *Toxomerus watsoni* (x2) is important in regulating the apterous (R^2^ = 0.0106) and alate aphid populations (R^2^ = 0.1033) in 0.40 m-spaced cotton. The *L. testaceipes* parasitoid (R^2^ = 0.0106) is also important in controlling alate aphids under such conditions. With respect to cotton grown under conventional spacing (0.80 m), in addition to *S. rubicundus* (R^2^ = 0.9132 for apterous and R^2^ = 0.8363 for alate aphids), the natural enemies *L. testaceipes* (R^2^ = 0.0092 for apterous and R^2^ = 0.0382 for alate aphids) and *C. sanguinea* (R^2^ = 0.0119 for apterous and R^2^ = 0.0222 for alate aphids) are relevant in controlling the populations of apterous and alate individuals. For the 1.60 m spacing, *C. externa* (R^2^ = 0.3101) was the natural enemy that along with *S. rubicundus* (R^2^ = 0.5132), constituted the multiple regression equation that was able to predict the number of apterous aphids, whereas *S. rubicundus* (R^2^ = 0.4569), *C. externa* (R^2^ = 0.3015) and *T. watsoni* (R^2^ = 0.0654) were considered the most important biological control agents responsible for the variation in alate aphid populations (Table 3).

**Figure 4:**
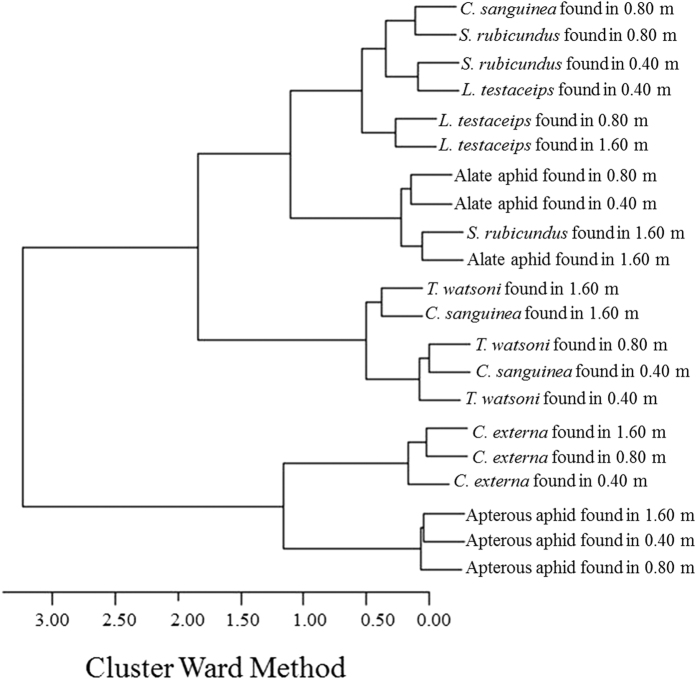
Co-occurrence of apterous and alate aphid morphs and their natural enemies at different spacings between cotton rows.

The Jaccard distance matrices allowed the investigation of insect groupings through the Ward method. The Jaccard dissimilarity coefficient was evaluated based on the binary data for plants with and without the presence of insects in each collection year; the codes 0 and 1 were attributed to the absence and presence of insects, respectively. This method allowed the comparison of closer or more distant occurrences between the apterous and alate forms and their natural enemies. By analyzing the dendrogram from the left to the right and by inserting a cut at approximately 2.00, it is possible to see two large and well-defined groups in the clusters based on the sums of squares using the Ward method. These sums show how much the presence or absence of apterous aphids in the cotton crops resemble the pattern observed for *C. externa* in the three cotton crops that used three different cotton crop row spacings (Fig. 4).

By checking evaluating the subgroups within the dendrogram from the base to the apex, it appears that the occurrence of alate aphids in the three cotton crops that have adopted the three different row spacings was very similar to the occurrence of *S. rubicundus* in the 1.60 m spacing. In addition, a high degree of similarity was found in the occurrence of *S. rubicundus* in cotton crops with 0.80 and 0.40 m spacing compared to the occurrence of *C. sanguinea* in cotton cultivations using a 0.80 m row spacing and to the occurrence of *L. testaceipes* in the three cropping systems that have adopted the three different row spacings. By following the same procedure, it is clear that the occurrence of *T. watsoni* populations in cotton crops that used the three different row spacings were grouped with the occurrence of *C. sanguinea* populations in cotton crops that used the 0.80 and 1.60 m row spacings. In addition, these were the most distant species within this large group (Fig. 4).

## Discussion

Our results showed that fluctuations in the populations of aphids and natural enemies as a function of time depend on the cotton cultivation year; however, the effect of crop age does not depend on crop spacing. In fact, crop age is a very important factor in the colonization by and outbreaks of *A. gossypii* populations in cotton crops^5^,^6^. According to De Bortoli *et al*.^23^, the age of the cotton plant was an important factor in determining the effects of biological control agents against *A. gossypii* on cotton grown at two different spacings at two different locations in Mato Grosso State, Brazil. However, the authors did not find that the effect of plant age on the action of natural enemies against aphids was dependent on row spacing. A reduced or increased spacing between cotton rows led to differences in the occurrence of multiple variables (alate and apterous aphids and their natural enemies) in relation to the conventional spacing between cotton rows (0.80 m); however, there is strong evidence that these differences do not occur between the 0.40 and 1.60 m row spacings.

The degree of the response of each variable as a function of time and of each cotton row spacing revealed by biplot charts, Pearson’s correlation and canonical analyses allowed confirm that the occurrence of *S. rubicundus* is directly related to the presence of apterous and alate aphids, as shown in all biplot charts. The high Pearson’s correlations between *S. rubicundus* and apterous and alate aphids may be explained through the occurrence of *A. gossypii* and its aggregation levels. In fact, Silva *et al*.^24^ found that the aggregation of such aphids on cotton grown in irrigated or rainfed systems increased with mean infestation, although it did not reach extreme values.

With the differences in the composition of principal components it was possible to recognize the importance of the *S. rubicundus* ladybug in the population control of apterous and alate *A. gossypii* at all the spacings used in the current study. The importance of such coccinellids has also been reported in fennel culture systems intercropped with colored fiber cotton in the Northeast region of Brazil^3^ because they have also contributed to reducing attack by *A. gossypii* and, consequently, damage to the cotton crop^6^. Yábar *et al*.^25^ used a multivariate approach to conduct a study of the culture of *Chenopodium quinoa* Willd. The authors observed that the temporal population dynamics of Aphididae, Coccinellidae and Braconidae resemble the traditional dynamics of natural enemies and pests.

The stepwise multivariate multiple regression analysis shows that *S. rubicundus* was the only independent variable found in all models (spacing/apterous/alate) to predict the occurrence of apterous and alate aphids. This selection procedure shows at most two variables that are responsible for regulating the apterous aphid populations at both the 0.40 and 1.60 m spacings. For the alate aphids, in addition to the two variables selected for apterous aphids, another variable was found for both spacings; thus, the parasitoid *L. testaceipes* and the predator *T. watsoni* are relevant to alate aphid control at the 0.40 m and 1.60 m spacings, respectively. Therefore, the variable selection method allowed for the highlighting of information of crucial importance to *A. gossypii* population control in cotton cultivation in the presence of alate individuals because the occurrence of such individuals is often associated with a high population density of the plants. The stepwise selection method allowed the reassessment at each phase or stage of analysis of the role played by the variables incorporated during the earlier stages. Such reassessment using the partial F tests emphasized that changing the row spacing structure also changes the importance of the natural enemies responsible for the population variation in both forms of *A. gossypii*. According to the quantitative analysis, *S. rubicundus* and the natural enemies *L. testaceipes* and *C. sanguinea* are important in controlling the populations of individuals on cotton grown under conventional spacing (0.80 m). In addition, there was no distinction in the composition of these variables between apterous and alate insects.

On cluster analysis, it is clear that *C. externa* populations are grouped with apterous aphids at the three spacings. The occurrences of alate aphids at the three spacing levels are very similar to that found for *S. rubicundus* at the 1.60 m spacing. The results of the current study show through patterns in similarity that the co-occurrence of apterous and/or alate aphids and their natural enemies within the same cropping system responds to variations in the spatial arrangement and results in more positive than negative or random associations^4^. However, a similar distribution does not always reflect a positive association between insect species within the culture system. In fact, the application of temporal distribution models to some natural enemies such as *L. testaceipes* and *S. rubicundus* should not be based on the absence and presence of insects, regardless of the cotton culture system (unpublished data). Thus, such information should be meticulously assessed. Therefore, it was concluded that similarities in the occurrences of apterous and/or alate aphids and their natural enemies respond to variation in the spatial arrangement mediated by the spacing between rows as do the variation in populations over time and the selection of important variables to regulate aphid populations. Such information is of great importance to develop strategies to control the *A. gossypii* population in these culture systems. It is worth highlighting the optimization of aphid sampling strategies and the conservation and release of natural enemies.

## Methods

### Study location and cotton cultivar

The study was conducted at Embrapa Cotton Experimental Station, Campina Grande County, Paraíba State, Brazil, at 550 m altitude and 7°13′11″ S latitude, 35°52′31″ W longitude. The cotton cultivar used in the current study was BRS 286. Cotton was planted on dark latossoil, under dryland conditions. The field plots were planted in the first week of March in 2013 and between the second and third weeks of March in 2014. Weed control was performed by hoeing.

### Experimental design

A randomized block experimental design was used that included three treatments of the following spacings: S1: 0.40 m × 0.20 m, S2: 0.80 m × 0.20 m, and S3: 1.60 m × 0.20 m, which were distributed in four replicates. The area of each experimental unit was 640 m^2^. Ten plants per meter of row were kept after thinning. The cotton plots were not sprayed with any insecticide to allow for natural aphid infestation, along with their predators and parasitoids. Each plant assessed was selected at random from each experimental unit.

### Arthropod specie sampling

The insects’ distribution and population dynamics on the cotton plants were determined at intervals of seven days and began when the plants emerged. The assessments were performed for 15 plants per plot. Insect quantification and their specific locations were recorded by taking as a reference the locations of the nodes of the main stem of the plant (from node zero to the terminal node) as well as the leaves and fruiting structures. Each plant was divided into three regions to facilitate the quantification of insects (apterous and alate forms of *A. gossypii* and its natural enemies), namely: basal (lower third of the plant), medial (mid third of the plant) and apical (upper third of the plant); however, the analyses considered the sum of the three regions.

### Data analysis

In the analyses, the dependent variables were abundance of apterous and alate forms of *A. gossypii, S. rubicundus, T. watsoni, L. testaceipes, C. sanguinea, C. externa.* The independent variables were spacings, assessment time and year. To test the hypotheses about influence of spacings, year and sampling week on insects abundance, we analyzed data from 2 years (2013 and 2014), using a Multivariate Analysis of Variance (MANOVA). The MANOVA produced an overall model that examined the effects of each independent variable and their interactions. If the overall model showed significant effects (main effects or interactions), we further examine each interactions or isolated effect using contrasts. The data were transformed using the Box-Cox method and MANOVA was performed using the Proc GLM procedure in SAS^26^ to meet the prerequisites of the multivariate variance analysis, i.e., the data exhibit a multivariate normal distribution and homoscedasticity of the variance and covariance matrices. The multivariate statistical tests Wilks’ Lambda, Pillai’s Trace and the Hotelling-Lawley Trace were used.

To identify major patterns of variation and ordination of the aphids and theirs natural enemies occurrence with respect to independent variables mentioned we conducted principal component analysis (PCA). To each cotton spacing was performed one PCA. Regarding the principal components analysis, the data were standardized by dividing the difference between each data point and the arithmetic mean of the variable of interest by the standard deviation of the variable. The relationships between variables pests (aphids alate and apterous) and natural enemies (*S. rubicundus, T. watsoni, L. testaceipes, C. sanguinea, C. externa*) were examined by canonical correlation analyses. The principal component analysis and the Pearson’s and canonical correlations were performed using the following procedures in SAS^26^: Princomp, Corr and Cancorr, respectively.

A stepwise (both forward and backward) multiple regression was performed to determine the impacts of natural enemies on aphids abundance. The decision threshold to include a given independent variable in each regression was based on p < 0.15. In the first run-through of the stepwise regression the species *S. rubicundus, T. watsoni, L. testaceipes, C. sanguinea, C. externa* were used as candidate independent variables to explain the variation of aphids abundance in each cotton spacing. The stepwise analysis was conducted using Proc Reg (selection = Stepwise)^26^.

In addition, the dissimilarities in the occurrence of aphids and their natural enemies among the different spacings in both years were determined using hierarchical cluster analysis with CLUSTER and TREE procedures^26^. Ward’s method was calculated for presence/absence data of insects (aphids and natural enemies) to examine the co-occurrence in all spacing. Ward’s method was used as a clustering technique to identify the zones in data and Jaccard distance was used as a distance matrix.

## Additional Information

**How to cite this article**: Malaquias, J. B. *et al*. Multivariate approach to quantitative analysis of *Aphis gossypii* Glover (Hemiptera: Aphididae) and their natural enemy populations at different cotton spacings. *Sci. Rep.* **7**, 41740; doi: 10.1038/srep41740 (2017).

**Publisher's note:** Springer Nature remains neutral with regard to jurisdictional claims in published maps and institutional affiliations.

## Figures and Tables

**Table 1:** Summary of the MANOVA results regarding the effects of spacing between cotton rows, time of assessment and year on the occurrence of cotton aphids and their natural enemies.

Source	Df	Wilks’ Lambda	Pillai’s Trace	Hotelling-Lawley Trace
		F value; P > F	F value; P > F	F value; P > F
Spacing (S)	14	0.9807	0.01934	0.01957
		3,174.00; = 0.0057	3,174.00; = 0.0057	2,535.80; = 0.0057
Assessment (time) (A)	91	0.5762	0.5010	0.6107
		10.06; <0.0001	9.45; <0.0001	10.64; <0.0001
Year (Y)	7	0.8905	0.1094	0.1229
		27.87; <0.0001	27.87; <0.0001	27.87; <0.0001
Block	21	0.9513	0.0491	0.0506
		3.80; <0.0001	3.78; <0.0001	3.83; <0.0001
S × A	182	0.8789	0.1275	0.1306
		1.14; = 0.1005	1.14; = 0.1010	1.14; = 0.1004
S × Y	14	0.9933	0.0066	0.0066
		0.75; = 0.7197	0.76; = 0.7193	0.75; = 0.7201
A × Y	91	0.6051	0.4627	0.5476
		9.13; <0.0001	8.67; <0.0001	9.54; <0.0001
S × A × Y	182	0.8943	0.1105	0.1127
		0.98; = 0.5509	0.98; = 0.5491	0.98; = 0.5525

The data were transformed according to the Box-Cox method as a prerequisite of the multivariate analysis of variance.

**Table 2:** Canonic correlations between the variables and the groups W_1_, W_2_ and V_1_, V_2_ at different spacings between cotton rows.

Variables	Spacing (m)/group 1			Spacing (m)/group 2		
	0.40	0.80	1.60	0.40	0.80	1.60
Correlations between the variables of group “V” and the canonical variables of group “W”						
		**V**_**1**_			**V**_**2**_	
Apterous	0.9497	0.9655	0.9087	−0.1314	0.0617	−0.0818
Alate	0.9031	0.9317	0.9049	0.2347	0.1708	0.0905
Correlations between the variables of group “W” and the canonical variables of group “V”						
		**W**_**1**_			**W**_**2**_	
*L. testaceipes*	0.1639	0.1902	0.1215	−0.2979	0.5310	0.1201
*C. externa*	−0.0869	− 0.0525	−0.1208	0.0195	−0.0131	−0.0363
*S. rubicundus*	0.9436	0.9648	0.7115	−0.1233	−0.0421	−0.0936
*C. sanguinea*	0.1601	0.5469	0.1006	−0.3384	−0.2413	−0.0506
*T. watsoni*	0.5666	−0.0309	0.0970	0.3106	0.3857	0.4233
Canonic correlation values						
	Spacing (m)					
*Canonic component*	0.40		0.80		1.60	
*1*	0.9701		0.9704		0.9255	
*2*	0.6426		0.6101		0.4301	

Wilks’ Lambda (F = 34.73, df = 10, den df: 154), Pillai’s Trace (F = 19.05, df = 10, den df: 156), Hotelling-Lawley Trace (F = 57.59, df = 10, den df: 112.78) and Roy’s Greatest Root tests (F = 113.25, df = 5, den df: 78) were significant (P > F < 0.0001). Variables of canonical group “V” are: alate and apterous aphids, and the variables of group “W” are *L. testaceipes, C. externa, S. rubicundus, C. sanguinea* and *T. wat.*

**Table 3:** Estimated coefficients of the stepwise linear regression analysis of apterous and alate aphids at different spacings between cotton rows.

Spacing of rows (m)	Aphid morph/NE species*		Partial R^2^	Model R^2^	C(p)	F Value	P > F
0.40	Apterous	*S. rubicundus (x*)	0.9003	0.9003	3.1124	234.85	<0.0001
		*T. watsoni (y*)	0.0106	0.9110	2.2183	2.99	0.0962
	Alate	*S. rubicundus (x*)	0.6945	0.6945	27.99	59.11	<0.0001
		*T. watsoni (y*)	0.1033	0.7978	12.41	12.77	0.0015
		*L. testaceipes (z*)	0.0632	0.8610	3.66	10.91	0.0030
0.80	Apterous	*S. rubicundus (x*)	0.9132	0.9132	5.8442	273.58	<0.0001
		*C. sanguinea (y*)	0.0119	0.9251	3.7514	3.97	0.0572
		*L. testaceipes (z*)	0.0092	0.9343	2.5859	3.36	0.0791
	Alate	*S. rubicundus (x*)	0.8363	0.8363	11.04	132.82	<0.0001
		*L. testaceipes (y*)	0.0382	0.8745	4.87	7.60	0.0107
		*C. sanguinea (x*)	0.0222	0.8966	2.13	5.14	0.0326
1.60	Apterous	*S. rubicundus (x*)	0.5132	0.5132	39.91	27.40	<0.0001
		*C. externa (y*)	0.3101	0.8232	1.20	43.86	<0.0001
	Alate	*S. rubicundus (x*)	0.4569	0.4569	45.05	21.87	<0.0001
		*C. externa (y*)	0.3015	0.7584	8.72	31.20	<0.0001
		*T. watsoni (z*)	0.0654	0.8238	2.40	8.92	0.0064

*NE: natural enemy. All variables remaining in the model are significant at the *0.1500* level.

## References

[b1] Carvalho L. H. & Chiavegato E. J. Semeadura adensada incrementa produção e reduz custos. Visão Agrícola: Algodão: melhores preços levam à ampliação da área cultivada (USP ESALQ, ano 3, Jul/Dez, 2006).

[b2] Kappes C., Zancanaro L. & Francisco E. A. B. Nitrogen and potassium in narrow-row cotton. Rev Bras Cienc Solo. 40, e0150103 (2016).

[b3] Ramalho F. S., Fernandes F. S., Nascimento A. R., Nascimento Júnior J. L., Malaquias J. B. & Silva C. A. Assessment of fennel aphids, *Hyadaphis foeniculi* (Passerini) (Hemiptera: Aphididae) and their predators in fennel intercropped with cotton with colored fibers. J Econ Entomol. 105, 113–119 (2012a). 22420262 10.1603/ec11219

[b4] Fernandes F. S., Malaquias F. B., Godoy W. A. & Santos B. D. Interspecific associations between *Cycloneda sanguinea* and two aphid species (*Aphis gossypii* and *Hyadaphis foeniculi*) in sole-crop and fennel-cotton intercropping systems. Plos One. 10, e0131449 (2015). 26241862 10.1371/journal.pone.0131449 PMC4524726

[b5] Fernandes F. S., Ramalho F. S., Malaquias J. B., Nascimento Júnior J. L., Correia E. T. & Zanuncio J. C. Within-plant distribution of cotton aphid (Hemiptera: Aphididae) in cotton cultivars with colored fibers. An Acad Bra Ciênc. 84, 707–719 (2012). 10.1590/s0001-37652012005000040 22782536

[b6] Ramalho F. S., Fernandes F. S., Nascimento A. R. B., Nascimento Júnior J. L., Malaquias J. B. & Silva C. A. D. Feeding damage from cotton aphids, *Aphis gossypii* Glover (Hemiptera: Heteroptera: Aphididae), in cotton with colored fiber intercropped with fennel. Ann Entomol Soc Am. 105, 20–27 (2012b).

[b7] Ramalho F. S., Malaquias J. B., Brito B. D. dos S., Fernandes F. S. & Zanuncio J. C. Assessment of the attack of *Hyadaphis foeniculi* (Passerini) (Hemiptera: Aphididae) on biomass, seed and oil in fennel intercropped with cotton with colored fibers. Ind Crop Prod. 77, 511–515 (2015).

[b8] Bachmann A. C., Nault B. A. & Fleischer S. J. Alate aphid (Hemiptera: Aphididae) species composition and richness in northeastern USA snap beans and an update to historical lists. Fla Entomol. 97, 979–994 (2014).

[b9] Andress M. Q. & Gould J. Multivariate analysis of *Bemisia tabacia* and parasitoid populations. In Gould J., Hoelmer K. & Goolsby J. Classical biological control of *Bemisia tabaci* in the United States: A review of interagency research and implementation, p. 287–306 (Springer, New York, 2008).

[b10] van Wingerden W. K. R. E., Griffioen A. J., van der Veen M., van der Straten M. J. J., Noordam A. P., Heijerman Th., ter Braak C. J. F. Meeuwsen H. A. M., Timmermans H. & Bianchi F. J. J. A. Effects of green veining on natural enemies of invertebrate pest species in leek and sprouts. Proc Neth Entomol Soc. 15, 99–103 (2004).

[b11] Letourneau D. K. & Goldstein B. Pest damage and arthropod community structure in organic vs. conventional tomato production in California. J Appl Ecol. 38, 557–570 (2001).

[b12] Straub C. S., Simasek N. P., Gapinski M. R., Dohm R., Aikens E. O. & Muscella S. Influence of nonhost plant diversity and natural enemies on the potato leafhopper, *Empoasca fabae*, and pea aphid, *Acyrthosiphon pisum*, in alfalfa. J Pest Sci. 86, 235–244 (2013).

[b13] Schmalhofer V. R. Tritrophic interactions in a pollination system: impacts of species composition and size of flower patches on the hunting success of a flower-dwelling spider. Oecologia. 129, 292–303 (2001). 10.1007/s004420100726 28547608

[b14] Zheng F. Q., Zhang X. H., Qu C. H., Liu X. Q. & Qu S. J. Quantitative analysis of insect pest and natural enemy communities in Red Fuji apple orchard. Chin J Appl Ecol. 20, 851–856 (2009). 19565766

[b15] Lattin J., Carrol J. D. & Green P. E. Parte III correlação canônica. In: Análise de dados multivariados [tradução Harue Avritscher]. p. 255–286 (Cengage Learning, São Paulo, 2011).

[b16] Bouroche J. M. & Saporta G. L’Analyse des Données (Presses Universitaires de France, 1982).

[b17] Gonçalves A. C., Almeida R. M. V. R., Lins M. P. E. & Samanez C. P. Canonical correlation analysis in the definition of weight restrictions for data envelopment analysis. J. Appl. Stat., 10.1080/02664763.2013.772571 (2013).

[b18] Crowder D. W., Northfield T. D., Strand M. R. & Snyder W. E. Organic agriculture promotes evenness and natural pest control. Nature. 466, 10.1038/nature09183 (2010). 20596021

[b19] Nunes R. P. Métodos para a pesquisa agronômica (Centro de Ciências Agroveterinárias, 1998).

[b20] Patrick C. J., Cavanaugh K., Kototchick T. & Peter H. Quantifying co-occurrence patterns in space and time across aquatic systems with network analysis. Eco-DAS X. 1, 1–13 (2014).

[b21] Scheiner S. Manova multiple response variables and multispecies interactions. In Scheiner S. M., Gurevitch J. Design and analysis of ecological experiments. Second division p. 99–115 (Oxford University Press, New York, 2001).

[b22] Kaiser H. F. An index of factorial simplicity. Psychometrika. 39, 31–36 (1974).

[b23] De Bortoli S. A., Vacari A. M., Fernandes M. C., De Bortoli C. P., De Bortoli S. L. P. & Ramalho D. G. Efeito do espaçamento e de *Bacillus thuringiensis* Berliner sobre *Alabama argillacea* (Hübner), *Aphis gossypi*i Glover e inimigos naturais no algodoeiro. Comunicata Scientiae. 6, 202–211 (2015).

[b24] Silva G. F., Ramalho F. S., Pereira A. I. A., Nunes Júnior E. S. & Pereira R. G. Padrão de distribuição temporal de *Aphis gossypii* em algodoeiro irrigado e de sequeiro no estado do Ceará. Rev Ver Agroecol e Desenvol Sust. 5, 195–203 (2010).

[b25] Yábar E., Gianoli E. & Echegaray E. R. Insect pests and natural enemies in two varieties of quinua (*Chenopodium quinoa*) at Cusco, Peru. J Appl Entomol. 126, 275–280 (2002).

[b26] Sas Institute. SAS/STAT user’s guide (SAS Institute, 2006).

